# Vitamin K Prophylaxis in Newborns: A Narrative Review of the Molecular Basis, Clinical Evidence, and Comparative Effectiveness of Intramuscular Versus Oral Administration and Parental Hesitation

**DOI:** 10.3390/ijms27041669

**Published:** 2026-02-09

**Authors:** Annamaria Mirone, Debora Mannino, Roberta Leonardi, Caterina Carpinato, Carmine Mattia, Grazia Maria Palano, Nunzia Decembrino, Martino Ruggieri, Pasqua Betta

**Affiliations:** 1 Postgraduate Residency Program in Pediatrics, Department of Clinical and Experimental Medicine, University of Catania, 95123 Catania, Italy; annamariamirone98@gmail.com (A.M.); deboramannino1996@gmail.com (D.M.); 2 Neonatal Intensive Care Unit (NICU), A.O.U. Policlinico “G. Rodolico-San Marco”, P.O. “G. Rodolico”-University of Catania, 95123 Catania, Italy; g.mannino@tiscali.it (C.C.); lorettamattia@hotmail.com (C.M.); graziamaria.palano@gmail.com (G.M.P.); ninnidecembrino@gmail.com (N.D.); mlbetta@yahoo.it (P.B.); 3 Unit of Pediatric Clinic, A.O.U. Policlinico “G. Rodolico-San Marco”, P.O. “G. Rodolico”-University of Catania, 95123 Catania, Italy

**Keywords:** vitamin K deficiency bleeding, neonatal prophylaxis, intramuscular vitamin K, oral vitamin K, parental hesitation

## Abstract

Vitamin K prophylaxis represents a paradigmatic example of how molecular mechanisms directly translate into effective neonatal preventive care. In newborns, physiological immaturity of vitamin K metabolism, including limited placental transfer, low hepatic reserves, immature intestinal absorption, and dependence on vitamin K-dependent γ-carboxylation pathways, creates a unique vulnerability to vitamin K deficiency bleeding (VKDB). This narrative review integrates molecular and biochemical mechanisms with neonatal physiology and clinical evidence to examine the effectiveness of current prophylactic strategies. At the molecular and pharmacokinetic level, intramuscular (IM) administration ensures sustained bioavailability and reliable activation of vitamin K-dependent proteins, whereas oral regimens are more sensitive to formulation, dosing schedules, and absorption efficiency. Consistently, clinical and surveillance data demonstrate near-complete protection against both classic and late VKDB following IM prophylaxis, while oral approaches show greater variability, particularly in real-world settings. Importantly, increasing parental refusal of IM vitamin K undermines an intervention with well-established molecular efficacy, contributing to preventable severe bleeding events. By linking mechanistic foundations to clinical outcomes and implementation challenges, this review provides a translational framework for clinicians, researchers, and policymakers aiming to optimize neonatal vitamin K prophylaxis in contemporary practice.

## 1. Introduction

Vitamin K is an essential cofactor for the γ-carboxylation of specific glutamic acid residues in coagulation factors II (prothrombin), VII, IX, and X, as well as in proteins C and S. This biochemical process is indispensable for maintaining effective hemostasis [1].

This group of fat-soluble compounds includes phylloquinone (vitamin K_1_), predominantly of plant origin, and menaquinones (vitamin K_2_), which are derived from bacterial or animal sources [2]. Although the daily requirement for vitamin K is extremely small, deficiency during the early days of life represents a clinically significant condition, as it can lead to severe hemorrhagic events, including intracranial bleeding [3].

In newborns, the risk of vitamin K deficiency is markedly increased due to several physiological and environmental factors: transplacental transfer is minimal, hepatic stores at birth are limited, and human milk contains very low concentrations of vitamin K (1–9 µg/L) [4]. Moreover, the intestinal microbiota, the main endogenous source of menaquinones, develops only gradually over the first weeks of life [1]. As a result, newborns are particularly vulnerable to vitamin K deficiency bleeding (VKDB), a hemorrhagic disorder classified into early, classic, and late forms according to the time of onset [1].

Universal intramuscular administration of vitamin K has been recommended by the American Academy of Pediatrics since the 1960s and has led to a marked reduction in VKDB in countries where it has been adopted [3]. This preventive measure reduced the incidence of VKDB from approximately 1700 cases per 100,000 untreated newborns to 1 case per 100,000 following prophylaxis [5].

However, the condition has not been completely eradicated. Clusters of VKDB cases persist, particularly in regions where prophylaxis is not routinely provided or is refused by parents [3]. Recent studies have documented that even in high-income countries, such as the United States, cases of intracranial hemorrhage secondary to vitamin K deficiency still occur, often among breastfed infants whose parents declined intramuscular administration at birth [3]. These observations were corroborated by Shearer and colleagues, who reported that VKDB remains a relevant cause of neonatal hemorrhagic morbidity even in industrialized nations, with a higher prevalence in certain Asian regions for reasons not yet fully understood [1].

Globally, epidemiological differences are influenced by multiple factors, including healthcare practices, infant feeding methods, and availability of oral or parenteral vitamin K formulations [6]. These international differences offer important context for understanding parental refusal and barriers to implementation.

A comprehensive understanding of the biological role of vitamin K and the mechanisms underlying its deficiency is therefore essential to promote adherence to neonatal prophylaxis recommendations.

Existing reviews on vitamin K prophylaxis tend to address clinical guidelines, epidemiological trends, or historical effectiveness separately, rather than providing an integrated perspective that also includes molecular mechanisms, neonatal physiological susceptibility, and real-world implementation challenges. Emerging population-based evidence indicates that this issue is dynamic rather than static. A large U.S. nationwide cohort study published in December 2025, including more than five million newborns, documented a significant and progressive increase in the proportion of infants not receiving intramuscular vitamin K prophylaxis over the past decade, underscoring parental refusal as a growing and measurable public health concern [7]. Despite this alarming trend, there is still a lack of comprehensive synthesis linking the molecular and biochemical pathways of vitamin K action with neonatal physiology, the comparative effectiveness of intramuscular versus oral regimens, and the clinical and societal implications of declining prophylaxis coverage. This narrative review aims to address the current absence of integrated, up-to-date analysis by connecting mechanistic insights with clinical evidence and implementation considerations. This offers a translational framework to inform clinical practice and parent–clinician communication.

In this context, the specific objectives of the review are as follows: examine the principal biochemical and pathophysiological aspects of vitamin K and its metabolism, describe the epidemiology and clinical manifestations of neonatal deficiency, discuss the efficacy and safety of postnatal prophylaxis, and highlight the importance of effective clinician–parent communication in preventing VKDB amid growing parental hesitancy.

## 2. Methods

This narrative review was conducted to integrate molecular, biochemical, and clinical evidence related to vitamin K prophylaxis in newborns. The literature search was performed across PubMed/MEDLINE, Scopus, and Web of Science, covering publications from database inception up to December 2025. A broad combination of search terms was used, including: “vitamin K”, “vitamin K prophylaxis”, “vitamin K deficiency bleeding”, “VKDB”, “newborn”, “neonate”, “intramuscular vitamin K”, “oral vitamin K”, “vitamin K cycle”, “*GGCX*”, “*VKORC1*”, and “parental refusal”.

Additional relevant articles were identified by screening the reference lists of key publications. Original research articles, randomized controlled trials, observational studies, systematic reviews, and international guidelines were considered. Only articles published in English were included. Given the narrative nature of the review, studies were selected based on relevance, scientific rigor, and their contribution to mechanistic or clinical understanding, and findings were synthesized and reported narratively.

## 3. Physiology and Molecular Basis of Vitamin K in Newborns

Vitamin K comprises a family of fat-soluble compounds sharing a 2-methyl-naphthoquinone nucleus, which differ in the length of their side chain. This group includes phylloquinone (vitamin K_1_), of plant origin (found in green leafy vegetables and vegetable oils), and menaquinones (vitamin K_2_), of bacterial or animal origin, also produced by the intestinal microbiota [6,8]. Absorption occurs in the small intestine through the action of bile salts. The vitamins are transported within chylomicrons and subsequently distributed via VLDL/LDL lipoprotein. Vitamin K_1_ is primarily concentrated in the liver, where it is utilized for the synthesis of coagulation factors. Vitamin K_2_ (especially MK-7 and MK-9) exhibits higher bioavailability and a longer plasma half-life (up to 72 h), which allows it to act on extrahepatic tissues such as bones, blood vessels, and the brain [8]. Vitamin K plays an essential role in the hepatic synthesis of coagulation factors II (prothrombin), VII, IX, and X, as well as of the anticoagulant proteins C, S, and Z. Since these factors are involved in both the extrinsic pathway (factor VII) and the intrinsic pathway (factors IX and X), the activity of vitamin K is indispensable throughout the entire coagulation process, extending to the common pathway (factor II) [9]. It is an essential micronutrient required for the post-translational modification of these proteins through the γ-carboxylation of glutamate residues, a reaction necessary for their activation. This modification enables the proteins to bind calcium ions (Ca^2+^) and anchor to the phospholipid surfaces of cell membranes, where the enzymatic activation of the coagulation cascade takes place [8,9]. In the absence of vitamin K, the non-carboxylated precursors—known as PIVKA proteins induced by vitamin K absence (PIVKA)—are synthesized in an inactive form, leading to impaired coagulation and an increased bleeding tendency [8,9].

Vitamin K acts as an essential coenzyme in the process of γ-carboxylation of glutamic acid residues present in specific proteins, a crucial step for their biological activation. This mechanism, which takes place in the rough endoplasmic reticulum, involves two main enzymes: γ-glutamyl carboxylase (*GGCX*) and vitamin K epoxide reductase (*VKORC1*) [8]. It should be noted that the central role of vitamin K in the γ-carboxylation of coagulation factors and its clinical consequences in vitamin K deficiency bleeding are supported by extensive human and clinical evidence. In contrast, the more detailed structural and conformational aspects of *GGCX* function derive primarily from experimental and in vitro studies. *VKORC1* initially reduces vitamin K, which is present in foods in its quinone form, to its active hydroquinone form (vitamin K hydroquinone). This active form is then utilized by γ-glutamyl carboxylase, which catalyzes the deprotonation of the γ-carbon of the glutamate residue and the incorporation of CO_2_, generating a reactive carbanion and leading to the formation of γ-carboxyglutamic acid (Gla) [10]. During the reaction, vitamin K hydroquinone is oxidized to vitamin K epoxide, which must then be recycled by *VKORC1* through sequential reduction, first to quinone and then back to hydroquinone. This vitamin K cycle allows for the continuous reuse of the cofactor, explaining the low physiological requirement for vitamin K [10].

*GGCX* is an integral membrane protein containing nine transmembrane helices (TM1–TM9) and a large luminal domain, which forms the recognition site for vitamin K-dependent proteins [11]. TM6 and TM7 are critical for shaping the vitamin K-binding pocket. A structural disulfide bond maintains the stability of the enzyme. Vitamin K-dependent proteins (such as osteocalcin) contain a short “propeptide” sequence that serves as a recognition signal. This propeptide binds to *GGCX* and facilitates the correct positioning of the substrate protein within the catalytic site. Upon propeptide binding, *GGCX* undergoes a conformational rearrangement that primes the enzyme for catalysis. Within *GGCX*, a hydrophobic pocket accommodates vitamin K: the polar headgroup of the vitamin anchors within the catalytic site, while its long isoprenoid tail inserts into a hydrophobic tunnel. Residues such as Met401, Met402, and Lys218 are essential for vitamin K binding and for the catalytic process. *GGCX* positions the substrate glutamate residue within a dedicated pocket. Lys218 initiates the reaction by activating vitamin K, His160 stabilizes the reactive intermediate, and additional conserved residues form a small “molecular gate” that directs the electron flow required for γ-carboxylation [11]. At the physiological level, this tightly regulated enzymatic process provides only a limited functional reserve. In newborns, who have low vitamin K stores and immature hepatic function, even modest reductions in vitamin K availability or γ-carboxylation efficiency can result in a disproportionate decline in functional coagulation factor activity.

Despite the essential role of *VKORC1* in several biological processes, its three-dimensional (3D) structure has not yet been experimentally resolved [12]. At the molecular level, *VKORC1* contains four highly conserved cysteine residues (C43, C51, C132, and C135). Cysteines C132 and C135 form the catalytic core of the enzyme, whereas C43 and C51 participate in the electron-transfer process that drives the redox reaction responsible for regenerating vitamin K into its active hydroquinone form [12].

Beyond coagulation, vitamin K exerts several important systemic functions that are mediated through both canonical γ-carboxylation and non-canonical, *GGCX*-independent mechanisms. These functions are related to bone metabolism, the nervous system, mitochondrial functionality, antioxidant role and inflammatory regulation.

Regarding bone metabolism, vitamin K acts as an essential cofactor for *GGCX*, which catalyzes the conversion of specific glutamate residues into γ-carboxyglutamate in a subset of vitamin K-dependent proteins. This post-translational modification markedly increases the affinity of these proteins for calcium ions and is indispensable for their biologic activity. In bone, *GGCX*-mediated carboxylation activates osteocalcin, enabling high-affinity binding to hydroxyapatite crystals and proper anchoring within the mineralized matrix, thereby supporting bone mineral density and microarchitectural integrity [13]. On the other hand, accumulation of undercarboxylated osteocalcin reflects suboptimal vitamin K status and has been associated with impaired bone quality and increased fracture risk [14]. Furthermore, vitamin K-dependent carboxylation activates matrix Gla protein (MGP), a potent local inhibitor of soft-tissue and vascular calcification [15]. Carboxylated, phosphorylated MGP can bind calcium and hydroxyapatite and prevent their deposition in the arterial wall, whereas its inactive dephospho-undercarboxylated form accumulates in states of vitamin K insufficiency and correlates with arterial stiffness and calcific burden [8]. Through the coordinated activation of osteocalcin and MGP, vitamin K contributes to resolving the “calcium paradox”, promoting calcium deposition in bone while limiting ectopic calcification in the cardiovascular system [15]. In the nervous system, vitamin K, particularly menaquinone-4 (MK-4), is highly represented, and it is now recognized as a key modulator of neuronal membrane biology. MK-4 participates in the biosynthesis and turnover of sphingolipids, including sphingomyelin and ceramides, which are critical constituents of neuronal and oligodendrocyte membranes and essential for myelination, axonal integrity, and synaptic function [10]. Experimental models have shown that alterations in vitamin K availability can modify cerebral sphingolipid profiles and are associated with changes in cognition and motor behavior, supporting a mechanistic link between vitamin K-dependent sphingolipid homeostasis and neurophysiological function [8]. In addition, accumulating data indicate that vitamin K exerts neuroprotective effects by mitigating excitotoxicity and attenuating pathways related to oxidative stress and neuroinflammation [16]. Additionally, beyond its role as a cofactor for *GGCX*, vitamin K, especially MK-4, can function as an alternative electron carrier in the mitochondrial electron transport chain and as a lipophilic redox antioxidant [8]. In cellular and animal models, MK-4 has been shown to accept and donate electrons within mitochondrial membranes, thereby supporting electron flux and limiting excessive production of reactive oxygen species [17]. This redox cycling capacity contributes to the preservation of mitochondrial membrane potential, reduction in lipid peroxidation, and protection of neurons and other cell types from oxidative injury under conditions of metabolic or inflammatory stress [10]. Vitamin K also modulates key intracellular signaling cascades involved in inflammation and apoptosis. In vitro and in vivo studies indicate that MK-4 can inhibit activation of the nuclear factor-κB (NF-κB) pathway, reducing nuclear translocation of NF-κB and downstream transcription of pro-inflammatory cytokines such as TNF-α and IL-6 [8]. In parallel, vitamin K has been reported to interfere with mitogen-activated protein kinase (MAPK) signaling, including p38 and JNK pathways, thereby attenuating stress-induced apoptosis and inflammatory responses in vascular, neuronal, and other cell types [10,16]. Through these combined actions on NF-κB and MAPK, vitamin K may exert broader cytoprotective effects that extend beyond its classical role in coagulation. Among these non-canonical functions, effects on coagulation-related pathways and early neurovascular stability are most directly relevant to neonatal outcomes, whereas roles in bone metabolism, mitochondrial function, and inflammation are more likely to influence health across the life course.

### 3.1. Neonatal Physiology: Limited Placental Transfer, Low Hepatic Stores, and Breast Milk Deficiency

Newborns are particularly vulnerable to VKDB due to several physiological factors, including the inefficient placental transfer of vitamin K during pregnancy and the reduced hepatic uptake resulting from the immature expression of lipoprotein receptors. As described by Shearer et al. [6], the fragility of vitamin K status in neonates arises from the convergence of physiological and molecular factors that are specific to the early infant period, rather than from dietary insufficiency alone. After intestinal absorption, vitamin K is transported in the bloodstream bound to chylomicrons (CM) and chylomicron remnants (CR), which are subsequently taken up by the liver through lipoprotein receptors, primarily the low-density lipoprotein receptor (LDLR) and the LDL receptor-related protein 1 (LRP1). In neonates, however, this mechanism is still functionally immature: hepatic receptors such as LDLR and LRP1 are not yet fully expressed or active, resulting in limited hepatic uptake of vitamin K. Consequently, hepatic stores of the vitamin are markedly low at birth [6]. Indeed, although the fetal liver already contains modest amounts of phylloquinone, neonatal hepatic stores are markedly lower than those of adults and are characterized by the near-complete absence of long-chain menaquinones (MK-7–13), which instead account for approximately 90% of hepatic reserves in adults. An additional limiting factor in neonatal vitamin K availability involves intestinal lipid processing and lipoprotein transport. The assembly and secretion of chylomicrons, essential carriers for the intestinal absorption of fat-soluble vitamins, depend on the activity of the microsomal triglyceride transfer protein (MTP), a protein located in the endoplasmic reticulum of enterocytes [18]. Reduced MTP activity impairs chylomicron secretion and consequently limits the systemic transport of fat-soluble vitamins, including vitamin K, providing an additional explanation for the heightened neonatal vulnerability to vitamin K deficiency. Moreover, in the intestinal tract of newborns, the microbiota is still sterile during the first days of life and therefore unable to produce endogenous menaquinones (vitamin K_2_). In adults, these bacterially synthesized menaquinones represent a significant source of vitamin K, contributing substantially to the body’s overall vitamin K pool [4,17]. Consequently, at birth, newborns have very limited hepatic stores of vitamin K and low vitamin K levels in cord blood. Also, vitamin K concentrations in umbilical cord blood are extremely low (<0.1 nmol/L), with a maternal–fetal gradient of 20:1–40:1, suggesting limited efficiency of placental transfer [19]. Breast milk also contributes to this condition, as vitamin K levels in human milk are significantly lower than those in formula milk (median 2.5 µg/L vs. 24–175 µg/L, respectively) [17], which is insufficient to meet the neonatal requirements in the absence of direct supplementation or maternal vitamin K intake [4]. These considerations, when considered together, clarify why neonatal vitamin K prophylaxis is both necessary and effective. Administering vitamin K intramuscularly overcomes the physiological limitations of intestinal absorption, lipoprotein-mediated transport and immature hepatic uptake. This establishes a tissue reservoir that maintains sustained vitamin K availability and supports adequate γ-carboxylation of coagulation factors during the critical period of neonatal vulnerability [6].

These factors together delineate a critical window of neonatal vulnerability. The combination of minimal placental transfer, low hepatic vitamin K stores at birth, and delayed establishment of endogenous vitamin K sources creates a narrow physiological margin in the early neonatal period. This is why effective prophylaxis must be administered immediately after birth and why strategies that rely on postnatal intestinal absorption are more vulnerable during the first weeks of life.

### 3.2. Molecular Determinants of Neonatal Vitamin K Insufficiency

A genetic component can significantly influence the metabolism and bioavailability of vitamin K. Mutations in genes involved in the vitamin K cycle have been associated with rare hereditary forms of congenital deficiency of vitamin K-dependent coagulation factors, vitamin K-dependent coagulation factor deficiency (VKCFD). In these rare autosomal recessive conditions, mutations primarily involve the *GGCX* gene (OMIM *137167; cytogenetic location: 2p11.2) and the *VKORC1* gene (OMIM *608547; cytogenetic location: 16p11.2) [20]. Patients with mutations in the *GGCX* gene present with VKCFD type 1, whereas those with mutations in the *VKORC1* gene exhibit VKCFD type 2 [21]. At a population level, however, these monogenic forms represent an exceptionally small proportion of neonatal VKDB, as most cases are driven by acquired factors such as insufficient prophylaxis, exclusive breastfeeding, or malabsorption and cholestatic conditions. Pathogenic variants in *GGCX* or *VKORC1* are therefore primarily relevant in rare, severe, or unexplained cases, particularly when bleeding occurs despite appropriate prophylaxis [21].

Recent structural studies have substantially elucidated the molecular mechanisms underlying hereditary vitamin K-dependent coagulation factor deficiency (VKCFD). In VKCFD type 1, several pathogenic mutations affect critical residues within the catalytic pocket of *GGCX* (His160, Tyr395, Asn159, and Arg436), disrupting glutamate recognition and thereby preventing γ-carboxylation [11]. Moreover, *GGCX* function relies on proper binding to the propeptide of vitamin K-dependent proteins and on the integrity of a structural disulfide bond (Cys99–Cys450) that stabilizes the enzyme; mutations affecting these regions further compromise enzymatic activity [11]. Many mutations responsible for VKCFD type 2 (F55, N80, and F83) disrupt the interaction of *VKORC1* with vitamin K and prevent its recycling to the active hydroquinone form. Catalytic residues such as C132/C135 and the electron transfer cysteines C43/C51 are indispensable, and their alteration markedly reduces enzymatic activity [12]. Therefore, in cases of unexplained vitamin K deficiency, genetic analysis should include, whenever possible, sequencing of the *GGCX* and *VKORC1* genes, along with interpretation of related pharmacogenetic polymorphisms [20]. Polymorphisms in the *VKORC1* gene, when present, may represent genetic determinants of reduced vitamin K availability or function, thereby contributing to an increased risk of deficiency and subsequent neonatal hemorrhage [22]. In parallel, several studies have also investigated whether polymorphisms or mutations in the *GGCX* gene may impair enzymatic function, thereby predisposing newborns to severe hemorrhagic manifestations. One of the most relevant contributions is the study by Vanakker et al., in which the authors identified a functional *GGCX* polymorphism associated with an increased risk of severe neonatal hemorrhage. These variants appear to reduce the efficiency of the γ-carboxylation process, thereby limiting the neonate’s ability to activate vitamin K-dependent coagulation factors and resulting in heightened hemostatic vulnerability [23]. In neonates, pharmacogenetics presents an additional layer of complexity. Beyond the genetic variability in genes encoding clinically relevant pharmacologic proteins, these proteins undergo rapid maturational changes during early life. A recent systematic review by Yalçin et al. (2022) showed that, in newborns, genotype does not reliably predict phenotype because the enzymes and receptors involved in drug metabolism and action have not yet reached full functional maturity [22]. In this systematic review, which evaluated studies regarding patients younger than 1 year of age, including neonates, it has been shown that *VKORC1* gene polymorphisms significantly influence the response to vitamin K antagonists and dose requirements. In pediatric patients, age, *VKORC1* genotype, and the presence of concomitant polymorphisms in CYP2C9 and CYP2C18 substantially contribute to the variability in anticoagulant response and INR, collectively explaining up to approximately 70% of the interindividual variability in dosing in multivariate models. These observations extend the relevance of *VKORC1* beyond rare monogenic deficiency, framing it within a broader developmental pharmacogenetic context in which genotype–phenotype relationships are dynamically modulated by neonatal maturation [22]. It analyzes the genes that can modify the effect and pharmacokinetics of drugs in neonates. Yalçin et al. therefore emphasized that, to fully understand the risk of functional vitamin K deficiency and neonatal hemorrhage, it is essential to consider both the genetic profile (including *VKORC1* and potentially *GGCX* polymorphisms) and the developmental maturity of neonatal metabolic pathways, which can substantially modify the phenotypic expression of these polymorphisms. In the end, from a clinical perspective, current evidence does not support routine pharmacogenetic screening to guide neonatal vitamin K prophylaxis. Universal administration, preferably via the intramuscular route, remains the most effective preventive strategy against VKDB. Nevertheless, genetic determinants should be considered in selected clinical scenarios, such as severe or recurrent bleeding despite appropriate prophylaxis, absence of malabsorption or cholestasis, or suggestive family history. In these contexts, targeted analysis of key genes involved in the vitamin K cycle (e.g., *GGCX* and *VKORC1*) may aid diagnostic clarification and clinical management. Looking forward, integrating developmental pharmacology with pharmacogenetics while accounting for rapid neonatal ontogeny and genotype–phenotype “phenoconversion” (defined as the age-dependent mismatch between genetic background and functional enzymatic activity in early life) [22] could allow us to stratify risk and take a personalized approach to prevention in the future. However, robust prospective validation is required before this can be implemented in a clinical setting.

The molecular targets discussed in this section were selected based on their direct involvement in the vitamin K cycle (*GGCX* and *VKORC1*), their demonstrated relevance in neonatal or pediatric settings, and their mechanistic plausibility in modulating vitamin K bioavailability, activation, or functional response during early life. These targets represent points at which genetic variability intersects with developmental immaturity, thereby influencing susceptibility to functional vitamin K insufficiency and hemorrhagic risk in newborns. Other molecular pathways involved in lipid metabolism, hepatic transport, or extrahepatic vitamin K-dependent processes were not discussed in detail, as current evidence does not directly link them to neonatal vitamin K deficiency bleeding or to clinically relevant failure of prophylaxis. Where available, data are largely derived from adult populations or experimental models and remain insufficient to support translational implications in the neonatal setting.

## 4. Vitamin K Deficiency Bleeding (VKDB)

Within this mechanistic framework, vitamin K deficiency represents the clinical consequence of impaired γ-carboxylation in the neonatal period, where molecular constraints and developmental immaturity converge to increase bleeding risk.

VKDB, previously known as the hemorrhagic disease of the newborn, is an acquired coagulopathy caused by reduced activity of vitamin K-dependent coagulation factors (II, VII, IX, and X), resulting in a marked bleeding tendency in newborns and infants [19]. The etiology of VKDB can be classified as primary or secondary. In the primary form, no specific cause can be identified other than exclusive breastfeeding, which provides insufficient amounts of vitamin K. The secondary form, on the other hand, is associated with additional factors that reduce the effectiveness or availability of vitamin K, such as malabsorption due to hepatobiliary or intestinal diseases, inadequate dietary intake, or the use of vitamin K-antagonist medications [24].

Based on the age of onset, VKDB is usually divided into three main categories:

Early VKDB (≤24 h of life): occurs within the first 24 h and is often associated with maternal exposure to medications that interfere with vitamin K metabolism (such as anticonvulsants, vitamin K antagonists, or antituberculosis drugs). The incidence of early-onset VKDB without prophylaxis is estimated from 250 to 1700 per 100,000 births [25].

Classic VKDB (2–7 days of life): typically occurs during the first week of life in newborns who have not received prophylaxis and are exclusively breastfed.

In the absence of vitamin K supplementation, the current incidence of classic VKDB is estimated from 10 to 35 per 100,000 births [17].

Late VKDB (2–12 weeks, up to 6 months in exclusively breastfed infants): represents the most severe form, often associated with cholestasis, malabsorption, or lack of vitamin K prophylaxis at birth [24,26]. Another contributing factor is exclusive breastfeeding, due to the low concentrations of vitamin K in human milk, whereas formula-fed infants are almost unaffected, since most formula milks are artificially fortified with vitamin K. Late VKDB affects between 10 and 80 infants per 100,000 live births, with a higher risk among exclusively breastfed infants and the highest incidence reported in Asian populations [17,24]. Reported VKDB incidence estimates should be interpreted with caution, as they are influenced not only by differences in surveillance systems and case definitions but also by underdiagnosis and misclassification of cases and variability in access to diagnostic resources and healthcare services.

Although VKDB is rare in absolute terms, with incidence typically expressed per 100,000 live births, the severity of its clinical manifestations, particularly intracranial hemorrhage, accounts for its disproportionate clinical and public health impact.

The clinical manifestations vary depending on the type and severity of the deficiency. Early VKDB presents with severe bleeding within a few hours after birth, including intracranial, gastrointestinal, abdominal, or thoracic hemorrhages. The prognosis of the early-onset form is generally poor, as up to 25% of cases involve intracranial hemorrhage [25,27]. Classic VKDB is characterized by bleeding from the skin, umbilical stump, injection sites, or the gastrointestinal tract; it rarely involves the central nervous system [26]. Late VKDB represents the most severe form, presenting with intracranial, gastrointestinal, and cutaneous hemorrhages. Its incidence ranges from 10 to 80 per 100,000 untreated births but is reduced to fewer than 3 per 100,000 in countries with universal prophylaxis programs [24]. Moreover, late VKDB is frequently associated with significant neurological morbidity, primarily due to the occurrence of intracranial hemorrhages [17]. Among newborns who survive intracranial hemorrhage, 40–55% develop permanent neurological damage, including: epilepsy/seizure disorders (up to 60–70% of survivors), psychomotor or cognitive delay (30–50%), cerebral palsy or hemiparesis (20–40%), and microcephaly or post-hemorrhagic hydrocephalus (5–15%) [17].

The diagnosis of vitamin K deficiency (VKD) requires a well-founded clinical suspicion and a careful evaluation of coagulation tests to identify any abnormalities consistent with this condition [6]. However, the diagnosis can be challenging, since vitamin K circulates at low concentrations and in different chemical forms, with half-lives and plasma levels that vary according to the individual’s lipid profile. In particular, diagnostic confirmation can be achieved by observing the normalization of PT/INR values within approximately 20–30 min after vitamin K administration [19]. It is important to emphasize, however, that normalization of PT/INR should not be regarded as a definitive diagnostic gold standard, as it primarily reflects hepatic synthesis of vitamin K-dependent coagulation factors and does not fully capture the functional reserve of the coagulation system or the residual bleeding risk, particularly in neonates [19]. In the absence of adequate vitamin K intake, the protein induced by vitamin K absence (PIVKA-II) becomes detectable in the blood. This protein disappears by the fifth day of life following the oral administration of 1 mg of vitamin K at birth [28,29].

The European and North American Societies for Pediatric Gastroenterology and Nutrition (ESPGHAN and NASPGHAN) currently recommend that all newborns receive one of the following prophylactic regimens: [26]

- Administer 1 mg of vitamin K (phylloquinone) intramuscularly (IM) at birth;
- Administer three oral doses of 2 mg: at birth, at 4–6 days, and again at 4–6 weeks of age;
- Administer 1 mg of oral vitamin K weekly for 3 months.

From a preventive perspective, current prophylactic strategies are highly effective in reducing the incidence of classic and late VKDB, which account for the majority of severe and preventable bleeding events. In particular, the administration of vitamin K intramuscularly provides near-complete protection against late-onset vitamin K deficiency bleeding (VKDB), including intracranial hemorrhage. On the other hand, early VKDB is less consistently preventable, as it is often related to maternal medication exposure during pregnancy [17,25]. Table 1 summarizes the main clinical subtypes of vitamin K deficiency bleeding (VKDB), highlighting differences in timing, risk factors, incidence, clinical severity, and preventability by prophylaxis.

## 5. Approaches to Vitamin K Prophylaxis

Hemorrhagic disease of the newborn (HDN) was first described in the literature as an entity by Townsend in 1894, although bleeding in the newborn had been described in detail long before that time. The understanding of neonatal hemorrhage was greatly enhanced when Paul Morawitz discovered the coagulation cascade in 1904. However, it was not until 1937 that Brinkhous et al. demonstrated hypoprothrombinemia in an infant with bleeding. In 1894, Paul Carnot described the hemostyptic ability of gelatin, and bleeding infants were soon treated with subcutaneous injections of the substance. Then, in 1905, Emile Weil demonstrated that small injections of fresh animal serum could control bleeding in hemophiliacs. Soon afterwards, bleeding neonates were also treated with subcutaneous injections of rabbit or horse serum. In 1908, Samuel Lambert successfully performed a blood transfusion on a critically ill infant using his father’s blood as the donor blood [30].

The modern era of understanding the importance of vitamin K began with Dam and Doisy being awarded the Nobel Prize in 1943 for their work on identifying and isolating the new vitamin. Initially, water-soluble vitamin K3 analogs were used, such as Synkavit; some cases of hemolytic anemia and kernicterus have been reported in premature infants who received excessive doses. Synkavit was withdrawn in 1961. The American Academy of Pediatrics (AAP) published its seminal paper on vitamin K and its use in pediatrics [31]. This report described HDN as a “hemorrhagic disorder of the first days of life caused by a deficiency of vitamin K and characterized by deficiency of prothrombin, proconvertin [Factor VII] and probably other factors.” This paper also recommended a single parenteral dose of 0.5 to 1.0 mg of vitamin K1 (phytomenadione) to all newborn infants as prophylaxis. With the etiology of HDN identified, the disorder is now known as vitamin K deficiency bleeding (VKDB).

As with other prophylactic measures for infants, opposition to vitamin K persisted despite the evidence, and the need for its universal use at birth was continually questioned. In 1992, Jean Golding, an epidemiologist from Bristol, reported an association between intramuscular, but not oral, vitamin K administration and childhood cancer. She concluded that it would be prudent to administer vitamin K orally rather than intramuscularly [32]. However, Golding’s findings were not confirmed by other studies, and the sensationalist media debate has undermined public confidence in vitamin K altogether.

**Figure 1:**
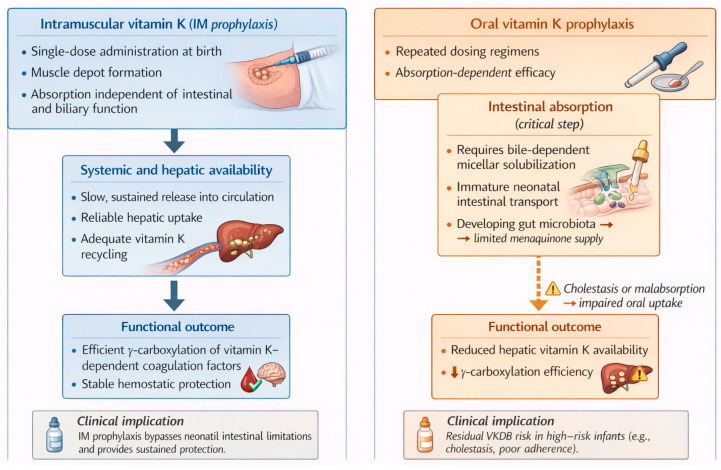
Mechanistic comparison between IM and oral vitamin K prophylaxis in newborns.

Vitamin K1 (phytomenadione) is a fat-soluble molecule; after IM injection, it is progressively released into the systemic circulation (Figure 1). There is no risk of poor absorption; it is independent of hepatobiliary disease (unlike the oral route) [33].

For example, Soedirman et al. demonstrated that, following intramuscular administration of 10 mg of phylloquinone in adult patients, the mean bioavailability of vitamin K1 is 82.9%, with a mean time to reach peak plasma concentration of 9.2 h. The mean peak plasma concentration is 67 ng/mL, and the area under the concentration–time curve (AUC) is 1700 ng/mL/h [34].

In babies given 1 mg of the vitamin orally at birth, the peak median concentration (73 ng/mL) occurred at four hours. By 24 h, median plasma concentrations had fallen to 23 ng/mL and 35 ng/mL in the groups fed vitamin K at birth or with the first feed, respectively; this difference was not significant. Plasma concentrations after an intramuscular injection exceeded those in the oral groups, with a peak median concentration of 1781 ng/mL at 12 h falling to 444 ng/mL at 24 h [35].

**Figure 2:**
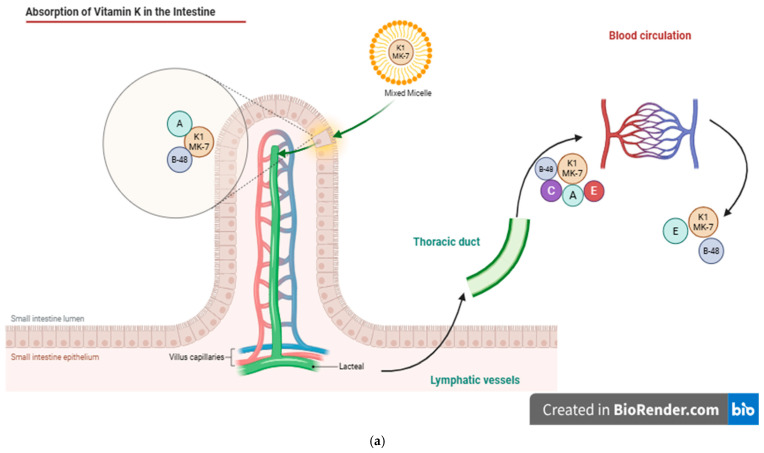

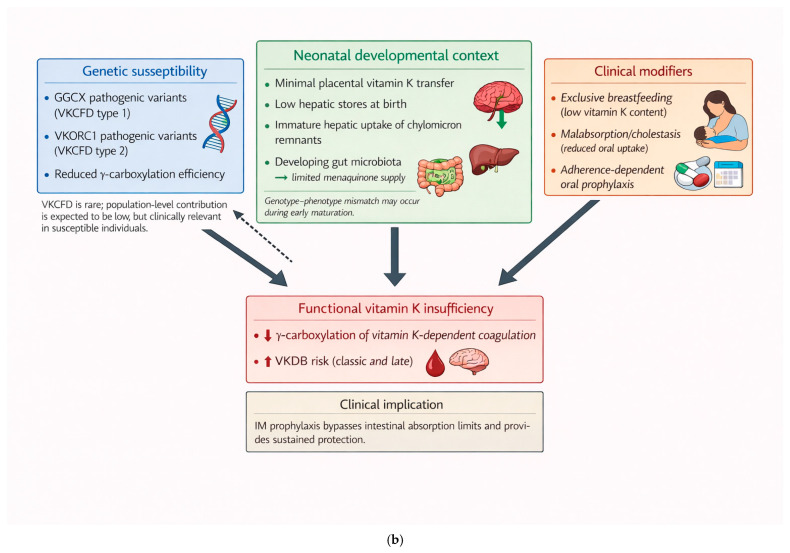
(**a**) The intestinal absorption of vitamin K. (**b**) Molecular and developmental determinants of functional vitamin K insufficiency in newborns.

Figure 2 shows the intestinal absorption of vitamin K (Figure 2a) and the mechanisms underlying vitamin K insufficiency in neonates (Figure 2b). As illustrated in Figure 2a, the intestinal absorption of phylloquinone is governed by the same principles established for other fat-soluble vitamins and highly lipid-soluble nutrients. In the intestinal lumen, vitamin K1 is packaged into mixed micelles composed of bile salts and the products of pancreatic lipolysis [36]. They are taken up by intestinal enterocytes of the small intestine and are incorporated into chylomicrons (CMs), which have apoA and apoB-48 on their surface; they are secreted into the lymphatic system and empty into the blood circulation via the thoracic duct. In the bloodstream, CM acquires apoC and apoE from HDL. CMs enter the capillary beds of peripheral tissues, where they lose apoA and apoC. The resulting chylomicron remnants (CRs) that reenter the circulation are smaller and have a central lipid core with surface apoB-48 and apoE [6]. The majority of vitamin K is delivered to the liver, and the uptake process is complex and involves different apoproteins. One important receptor component is apoE, a 34 kDa protein that is produced and secreted by many cell types, including hepatocytes, where direct secretion-capture removal of CRs takes place in the space of Disse [6].

Vitamin K1 is catabolized in the liver by a common degradative pathway in which the polyisoprenoid side chain is shortened to two major carboxylic acid aglycones with 7- and 5-carbon side chains, respectively, which are excreted in bile and urine mainly as glucuronides. The chemical structures of isolated metabolites are consistent with their formation by an oxidative degradation of the isoprenoid side chain by an initial ω-hydroxylation followed by a progressive side-chain shortening via the β-oxidation pathway. The cytochrome P450 enzyme, CYP4F2, carries out ω-hydroxylation [37].

Tracer studies with labeled K1 have shown that a sizable fraction of a single oral dose is rapidly catabolized in the liver and excreted predominantly in the bile, as well as in urine. This extensive catabolism of K1 by the liver explains the rapid turnover and depletion of hepatic reserves in patients on a low K1 diet. It has been estimated that 60–70% of the amount of K1 absorbed from each meal will ultimately be lost to the body by excretion. This suggests that the body stores of K1 are being constantly replenished [37].

Patients with biliary obstruction lack the detergent components of micelles, and the degree of absorption depends on the severity of the bile salt deficiency. In patients with chronic pancreatitis, the primary disturbance is a reduced generation of the solutes of mixed micelles, namely, 2-monoglycerides and fatty acids. This also impairs the absorption of phylloquinone, though this is usually less severe than in bile salt deficiency. The essentiality of bile salts to the absorption of vitamin K is reflected in the higher incidence of vitamin K deficiency in cholestatic disease compared to most other gastrointestinal diseases [36].

Table 2 summarizes the existing recommendations for the use of prophylactic vitamin K in newborns.

There is currently sufficient evidence demonstrating the effectiveness of vitamin K administration in preventing VKDB in newborns, and there is an international consensus regarding its administration to all infants. Nevertheless, there is no standardized dosage or administration method for all countries, as demonstrated by data from surveillance studies [38]. Further trials are needed to identify the best method and implement it worldwide to counteract divergences.

## 6. Comparative Effectiveness: Intramuscular Versus Oral Administration

Exclusive breastfeeding is considered a risk factor for VKDB, as human milk contains significantly lower VK concentrations (0.85–9.2 µg/L) compared to formula milk (4.24–175 µg/L) [41]. Perrone et al. demonstrated that the administration of VK prophylaxis through intramuscular injection and oral administration has been found to have similar effects on PIVKA-II (protein induced by vitamin K absence or antagonist II) concentrations at 48 h of life. It suggests that both intramuscular and oral intake of VK1 may be effective only in preventing classic VKDB, which can occur within the first week after birth. Additionally, routine oral supplementation of VK1 from discharge until 14 weeks of life, with a daily dosage of 150 micrograms, has been shown to significantly decrease PIVKA-II concentrations compared to a single intramuscular injection of 1 mg of VK1 at birth in exclusively breastfed term infants, preventing the risk of developing late VKDB [41].

This prospective multicentric observational study compared four treatment regimens, including those currently recommended by leading global organizations, as described in the table above. These findings present the first set of data reporting PIVKA-II assessment from birth until the third month of life, examining various regimens of VKDB prophylaxis [41]. PIVKA-II blood levels may be used as early markers of subclinical vitamin K deficiency at 30 and 90 days of life in exclusively breastfed newborns; despite high plasma levels of PIVKA-II not implying an impending occurrence of bleeding, they have been shown to be a sensitive alternative to PT/INR for assessing subclinical vitamin K deficiency [42].

This study has several limitations. Firstly, the sample size is small. Secondly, it only includes healthy term newborns without malabsorption problems. Thirdly, it relies on biochemical markers of vitamin K deficiency rather than assessing hemorrhagic outcomes directly. Finally, it only provides information about the first three months. While the results are limited, they could lay the foundation for further studies with a larger sample size, including children with malabsorption problems such as cholestasis. These results could lead to changes in current recommendations, making them uniform worldwide.

Cornelissen et al. showed that daily low-dose oral prophylaxis of 25 micrograms in breastfed infants following an initial dose of 1 mg after birth may be as effective as parenteral vitamin K prophylaxis [43]. This could indicate that the latter scheme of prophylaxis is as effective as intramuscular prophylaxis. But since these studies were not randomized controlled trials, their validity can be doubted. For example, the infants were born in different countries, and the participating countries observed the infants over different time periods between the 1960s and the 1990s. It is not known how this could have affected the results.

Croucher et al., in the *British Medical Journal*, demonstrated that after discharge, many newborns were not administered the oral doses provided by the country’s current recommendations [44]. Therefore, it is valuable to underline that non-adherence to recommended oral prophylaxis regimens could influence the data obtained from the studies in ideal conditions.

Sankar et al. conducted a systematic review to evaluate the burden of late VKDB and the effect of vitamin K prophylaxis on its incidence [45]. The median burden of late VKDB was 35 per 100,000 live births in infants who had not received prophylaxis at birth. They found only two randomized trials that evaluated the effect of intramuscular prophylaxis on the risk of classical VKDB. The first [46] reported a significant reduction in the incidence of any bleeding (RR: 0.73; 95% CI: 0.56 to 0.96) as well as moderate to severe bleeding (RR: 0.19; 0.08 to 0.46; NNT: 74, 47 to 177) following vitamin K3 prophylaxis (1 mg and 5 mg). In the second, Vietti et al. [47] demonstrated a significant reduction in the incidence of secondary bleeding after circumcision in neonates who received vitamin K prophylaxis (RR: 0.18, 0.08 to 0.42; NNT: 9, 6 to 15). The results of the two studies could not be pooled because of the different nature of the outcomes (spontaneous bleeding vs. post-circumcision bleeding).

Sankar et al. did not find any randomized or quasi-randomized trials that evaluated the effect of vitamin K prophylaxis on the incidence of late VKDB. Therefore, they used data from nationwide surveys conducted in four countries. The latter indicates that the use of IM/subcutaneous vitamin K prophylaxis could significantly reduce the risk of late VKDB when compared with no prophylaxis (RR: 0.02; 95% CI: 0.00 to 0.10); when compared with IM prophylaxis, a single oral dose of vitamin K increased the risk of VKDB (RR: 24.5; 95% CI: 7.4 to 81.0), but multiple oral doses did not (RR: 3.64; 95% CI: 0.82 to 16.3). This is low-quality evidence because of the lack of randomized controlled trials [45].

There are no randomized trials comparing the oral and intramuscular routes of vitamin K prophylaxis in newborns [38]. Jullien reported different regimens of vitamin K prophylaxis used in different European countries with the corresponding incidence of VKDB from surveillance data [38]. Mihatsch et al. concluded that while data from older studies suggest that IM may be more effective than the multiple oral doses of vitamin K for preventing late VKDB, the more recent data from surveillance systems do not seem to support a significant difference between the IM and the oral route for preventing late VKDB [26]. The efficacy of the oral regimen relies on compliance with the protocol.

Studies on the efficacy of treatments for premature infants are important because the main sources of phylloquinone for these babies during the neonatal period are the prophylactic dose given at birth and the phylloquinone derived from parenteral and/or enteral feeding [48]. The Ardell 2018 Cochrane review looked at the effect of vitamin K prophylaxis in the prevention of VKDB in preterm infants (gestational age < 37 weeks) [49]. No RCT looking at vitamin K via any route of administration versus no vitamin K was identified. Only one RCT comparing three arms of prophylactic vitamin K (0.5 mg IM, 0.2 mg IM, and 0.2 mg IV) in 80 preterm babies under 32 weeks of gestational age was identified [48]. There were no statistically significant differences between 0.2 mg IV versus 0.2 mg IM on bleeding complications (RR: 7.00 [95% CI: 0.38 to 129.11]), intraventricular hemorrhage grade II (RR: 2.00 [95% CI: 0.19 to 20.72]), presence of PIVKA-II at day 5 (RR: 1.52 [95% CI: 0.37 to 6.23]) or day 25 (RR: 1.08 [95% CI: 0.07 to 16.36]), necrotizing enterocolitis (RR: 1.00 [95% CI: 0.15 to 6.57]), or sepsis (RR: 1.00 [95% CI: 0.28 to 3.58]). There were no statistically significant differences between 0.2 mg IV and 0.5 mg IM on the same outcomes. When looking at higher (0.5 mg) versus lower (0.2 mg) doses of IM vitamin K, there were also no statistically significant differences with a broad 95% CI for the same outcomes. There are no randomized clinical trials that have determined the appropriate dose and route of administration of vitamin K in preterm infants; therefore, there are many opportunities for clinician researchers to design randomized clinical trials to appropriately study the dose and route of administration of vitamin K for preterm infants, particularly in preterm infants < 30 weeks’ gestation, where the risk of hemorrhagic complications (such as intraventricular hemorrhage) is high.

Preterm babies exclusively breastmilk fed should all be provided with additional daily oral VK1 or VK2 supplements routinely at hospital discharge. In fact, according to Clarke et al., without additional supplementation, these neonates are at a high risk of developing VK insufficiency in early infancy [50]. A recent research article described the probable role of lipid-soluble vitamins in protecting against brain injury in preterm infants [51]. The key targets of lipid-soluble vitamins are involved in processes related to glucose uptake across the plasma membrane, energy metabolism, ligand-receptor interactions of neuroactive substances, calcium ion signaling pathways, and regulation of the Hypoxia-Inducible Factor 1 (HIF-1α), a hypoxia-related pathway. Probably, 6-phosphofructo-2-kinase (PFKFB3) and platelet-derived growth factor receptor beta polypeptide (PDGFRB) are targets for vitamin E and vitamin K. Particularly, Lys318 and Asn321 on the PFKFB3 protein receptor formed hydrogen bonds, and Leu300 engaged in both hydrophobic and Pi-Sigma interactions with vitamin K; with regard to the PDGFRB protein receptor, Arg830 formed a hydrocarbon interaction, and residues Leu833, Cys684, Tyr683, Leu606, Val614, Tyr771, Arg849, and Val690 engaged in hydrophobic interactions [51]. Under physiological conditions, PFKFB3 protein is inactivated due to proteasomal degradation. However, it becomes stabilized and activated under excitotoxic stimuli, thereby impeding the redox state protection mediated by the pentose phosphate pathway (PPP) and leading to neurodegenerative changes. PDGFRB regulates the phenotypic transformation of vascular smooth muscle cells and neuroinflammation following intracerebral hemorrhage in mice. Therefore, PFKFB3 and PDGFRB are related to the occurrence and development of brain injury in preterm infants [51]. Li et al. utilized molecular docking simulations to investigate the binding mechanisms between vitamin K components and the identified key targets. The molecular docking results revealed that vitamin K–PDGFRB binding energy is 8.3034 kcal/mol, primarily driven by hydrogen bonds and hydrophobic interactions. Lower binding energies typically indicate stronger binding affinities between the small molecule active components and the target sites. These findings could suggest that vitamin K demonstrated stronger binding affinities for the key targets [51].

Molecular docking analyses provide a theoretical basis for screening potential targets and mechanisms, laying the groundwork for subsequent experimental validation. These computational methods used in the study cited above are valuable for generating hypotheses, but the predicted interactions and pathways may not fully reflect the complex biological reality. Future research should employ cell experiments and animal models to validate and explore the mechanisms by which this vitamin modulates brain injury-related pathways in preterm infants both in vitro and in vivo [51].

Hypoxia-inducible factor (HIF)-1α, which belongs to a transcription factor found in mammals and humans under hypoxic conditions, can upregulate the expression of numerous genes that are responsive to hypoxia and regulate the balance for cell activity under hypoxic conditions. Overexpression of HIF-1α or induction of HIF-1α activity can alleviate brain damage and facilitate neurological functional recovery. Lipid-soluble vitamins bound to PFKFB3 and PDGFRB may indirectly affect downstream of HIF-1α pathways [51]. These predicted interactions are hypothesis-generating and do not necessarily indicate a validated causal mechanism; they should be considered candidates for experimental validation.

The higher efficacy for prevention of late VKDB of intramuscular compared with a single oral dose of phytomenadione prophylaxis could be a result of poor absorption of phytomenadione administered orally or its depot effect at the site of injection [52].

Depot preparations are sustained-release pharmaceutical formulations designed to produce very slow absorption from the site of injection. This is commonly achieved by chemically altering a water-soluble drug to render it insoluble and then administering it as an aqueous suspension. The insoluble vitamin K1 must be solubilized using an agent. The chemical properties of vitamin K1 are such that it could readily form an aggregate or precipitate once mixing occurs with interstitial fluid in muscle. A portion of the injected vitamin rapidly enters the circulation [35] and is carried bound to lipoproteins. It is probable that, due to its extreme insolubility in water, the remaining portion of the injected vitamin may form a viscous mass in muscle tissue, which could then be slowly absorbed into the circulation over many weeks; thus, IM vitamin K1 may be an unintended depot preparation [53].

Cornelissen et al. compared plasma vitamin K1 levels after administering 1.0 mg of Konakion by either the IM or oral routes. The values at 1 month were 0.62 pg/L (IM) and 0.39 pg/L (oral), *p* < 0.0001; at 3 months, the values were 0.33 pg/L (IM) and 0.27 pg/L (oral), *p* = 0.03 [54]. The finding at 3 months of a small but significant difference is consistent with a depot effect and is difficult to explain on any other basis. Loughnan et al. reported the occurrence of late-onset hemorrhagic disease in two small premature infants who received 0.12 and 0.94 mg/kg of intravenous vitamin K1, respectively. The lack of a depot effect with intravenous vitamin K1 and its rapid hepatic turnover rate may explain the failure of intravenous vitamin K1 prophylaxis in these infants [55].

Harrington et al. measured the excretion of two urinary metabolites of vitamin K (5C- and 7C-aglycones) in term infants before and after a 1000 μg IM dose of vitamin K1 and in preterm infants after 200 μg IM, 500 μg IM, or 200 μg IV and demonstrated that urinary 5C- and 7C-aglycone excretion by term and preterm infants was dose dependent and increased within 24 h of K1 prophylaxis. The slower increase in excretion over 24 h after IM injection compared with an equivalent dose (200 μg) given by the IV route gives support to a depot effect whereby lipophilic vitamin K1 leaches out only slowly from the muscular site of injection into the circulation [56].

These findings confirm how essential it is to administer prophylaxis to all newborns to have long-term effects and ensure protection against VKDB. Further studies are necessary to provide more robust evidence and to determine the most effective and appropriate regimen for VK supplementation. In the meantime, for specific recommendations regarding VK prophylaxis and supplementation in infants, it is advisable to follow national and international guidelines based on the most up-to-date medical knowledge.

## 7. Safety and Adverse Events

### 7.1. Intramuscular Administration

Intramuscular administration can cause local trauma, injury to vessels and nerves, abscesses and muscle hematoma [45]. When the IM route with standard precautions at the anterolateral thigh is chosen, the risk of local reactions at the site of the injection is very low [38]. Although the IM injection is likely to be painful, simple strategies, including skin-to-skin, breastfeeding, or administration of glucose/sucrose, are effective to alleviate pain in newborns [57]. These nonpharmacologic strategies have been shown to be useful in decreasing pain scores during short-term mild to moderately painful procedures and should become part of routine neonatal comfort care [58].

The association of IM administration of vitamin K with certain forms of childhood cancer or any other severe side effect has been conclusively rejected by several studies [59,60,61,62,63]. Additionally, a recent systematic review and meta-analysis examined the impact of various early-life nutritional factors, including neonatal vitamin K administration, on the risk of childhood acute leukemia. The authors did not identify any significant association between vitamin K at birth and leukemia incidence, confirming previous findings and reinforcing the safety of this prophylaxis [64]. The literature has reported cases of anaphylactic shock and rare cases of Nicolau syndrome in premature infants following intramuscular administration [65,66,67]. In the first case, the newborn was a term male who was hospitalized with a diagnosis of transient tachypnea of the newborn and received prophylaxis with intramuscular injection of 1 mg vitamin K1 (Konakion). The formulation included polyoxyethylated castor oil (PEO-CO), used as a solubilizer or diluent for several drugs. Pereira and Williams observed that vitamin K1 formulated with PEO-CO was associated with more reactions than the MM formulation, suggesting that the solubilizer rather than vitamin K1 itself may be the causative agent [68]. This notion is also supported by case reports describing patients with previous reactions to medications containing PEO-CO [69]. In the review of Britt et al. [70], it was demonstrated that vitamin K1 may induce severe reactions, including death, when administered via the parenteral route, especially when given intravenously. These reactions are most consistent with an anaphylactoid mechanism. The majority of reactions have occurred in patients receiving vitamin K1 solubilized with PEO-CO, suggesting that the solubilizer may contribute to many of these reactions.

Nicolau syndrome is a rare complication of the intramuscular injection of various drugs. It has been reported in preterm infants with an extremely low birth weight following the intramuscular administration of 0.5 mg of vitamin K1, representing an epiphenomenon of injury to the blood vessels of the skin.

For over 50 years, the AAP has recommended prophylactic vitamin K to prevent hemorrhagic disease in newborns. In recent years, there has been an increasing trend of parental refusal and resultant bleeding [71].

Parents who refuse vitamin K prophylaxis made their decision well before delivery. Therefore, attempts to change their minds in the immediate postpartum period are unlikely to succeed.

Educating parents about the importance of IM prophylaxis should begin in the prenatal period and must address concerns parents identify on the Internet [72].

Primary medical providers, prenatal class instructors, and others concerned with newborn health must establish credibility with parents in prenatal settings through discussion and presentation of accurate information underlining the role of IM vitamin K in preventing late-onset VKDB [72].

The AAP has also published information to assist providers in addressing concerns about vitamin K [73].

Although prophylaxis against hemorrhagic disease of the newborn with vitamin K has been routine practice all over the world for many years, there are only a few reports in the literature of side effects after vitamin K injection in neonates.

The difference between the number of cases of adverse events and that of VKDB further underlines the importance of such prophylaxis.

### 7.2. Oral Administration

Oral route-specific risks are vomiting/spitting out and contraindication in biliary atresia because the reduced bile flow significantly affects the absorption of fat-soluble vitamins, including VK, leading to VK deficiency [38].

Witt et al. looked at the incidence of HDN in breastfed infants with biliary atresia who received one of the three following regimens of prophylactic vitamin K according to their country and date of birth: 1 mg orally at birth followed by 25 μg orally daily from week 2 to week 13 of life was given to infants born in the Netherlands from 1991 to 2011; 1 mg orally at birth followed by 150 μg orally daily from week 2 to week 13 of life was given to infants born in the Netherlands from 2011 to 2015; a single dose of 2 mg IM at birth was given to infants born in Denmark from 2000 to 2014. HDN occurred in 45/55 (82%) infants from the oral 25 μg group, in 9/11 (82%) of the 150 μg group, and in 1/25 (4%) of the 2 mg IM group, leading to the conclusion that an oral prophylaxis of 1 mg of vitamin K followed by a daily oral dose of 25 or 150 μg fails to prevent HDN in breastfed infants with unrecognized biliary atresia, in comparison to a single IM dose [74].

## 8. Strengths and Limitations of Available Evidence

The literature on vitamin K prophylaxis in newborns is extensive and generally consistent in its conclusions. A key strength lies in the convergence of evidence from multiple study designs, including randomized trials, systematic reviews, and large national surveillance studies. Across these sources, intramuscular (IM) vitamin K consistently demonstrates superior efficacy in preventing both classic and late VKDB, with reductions in incidence ranging from 96.7% to 98% compared with no prophylaxis [38,45]. Population-based data from Germany, the Netherlands, Australia, and the British Isles confirm nearly complete protection following IM administration, while oral regimens, particularly single-dose protocols, show higher rates of late VKDB [43,75]. The overall consistency across geographic and methodological contexts enhances the external validity of these findings. Another strength is the integration of biochemical and clinical data. Jørgensen et al. (1991) and Puckett and Offringa (2000) demonstrated comparable biochemical responses between oral and IM routes in the short term, lending biological support to clinical observations [76]. Furthermore, the safety of IM prophylaxis is well established. Concerns about potential cancer associations have not been substantiated, and adverse events are limited to rare local reactions [43]. Despite these strengths, it is important to acknowledge the limitations of the current literature, as these limitations influence the interpretation and generalizability of the findings. First, evidence comparing oral and IM regimens is largely derived from observational and surveillance data, which are prone to confounding and variable diagnostic definitions. Only a few randomized trials exist, and they often include small sample sizes or short follow-up [76]. Second, the literature on oral vitamin K prophylaxis is characterized by substantial heterogeneity. Studies differ widely in formulations, dosing schedules, duration of administration, and assessment of adherence, making direct comparisons across studies difficult and limiting the ability to identify an optimal oral regimen [43,45]. Moreover, several studies rely on biochemical surrogate markers rather than clinical bleeding outcomes, limiting their applicability to real-world prevention [33]. Finally, data remain scarce for specific populations, such as preterm or medically fragile infants, and for settings with limited healthcare access, where adherence to multi-dose oral regimens is challenging [77].

## 9. Parental Hesitation Currents

In recent years, there has been a growing trend of parental hesitation or refusal regarding the prophylactic IM administration of vitamin K to newborns, a practice internationally recommended to prevent VKDB. Refusal has been most studied and reported in Anglo-American contexts, but related hesitancy and barriers occur elsewhere and may be under-reported. In Europe and the rest of the world, prophylaxis is considered an integral part of standard neonatal care [5,78,79]. A key contribution is the international cross-sectional survey by Sirachainan et al. (2025) [80]. This survey assessed the current incidence and risk factors of VKDB and examined neonatal vitamin K prophylaxis practices. It also identified obstacles and limitations to the administration of prophylaxis in 38 countries within different healthcare settings. In high-income countries (Europe and North America), the main barrier was found to be parental hesitancy of the intramuscular injection, whereas in middle- and low-income countries, the predominant issues were drug scarcity and inadequate healthcare training, particularly among midwives in the administration of vitamin K [80]. The reasons underlying parental hesitancy are multifactorial and reflect an interplay of cultural, psychological, and communicative factors, including misinformation and distrust. In a large retrospective cohort study including more than five million newborns born between 2017 and 2024, Scott et al. (2025) [7] documented a steady increase in the number of babies not receiving intramuscular vitamin K, rising from 2.9% to 5.2%. This trend began prior to the onset of the global pandemic. The lack of significant changes in the clinical characteristics of newborns suggests that this increase is indicative of a wider trend of parental hesitancy towards preventive interventions rather than being driven by clinical factors. Moreover, the higher frequency of non-receipt among infants born vaginally appears consistent with birth care models oriented toward low levels of medical intervention [7].

Parental hesitancy of vitamin K prophylaxis seems to be characterized by different reasons and patterns. Particularly, it is generally motivated by concerns related to injection-associated pain, perceived invasiveness, or the presence of excipients, rather than opposition to vitamin K itself [78]. Another recurring motivation is the pursuit of “naturalness,” understood as the desire to preserve childbirth and the newborn’s first moments of life from interventions perceived as artificial or invasive [78]. In many cases, these fears are amplified by anecdotal accounts circulating on social media. These accounts contribute to the reinforcement of misconceptions and the spread of distrust towards medical recommendations [81,82]. In these cases, adherence to multi-dose oral regimens and the implementation of structured follow-up are essential to mitigate the increased risk of late VKDB. Second, a smaller group of parents refuses all forms of vitamin K prophylaxis. This behavior is more frequently associated with strong ideological positions, widespread distrust of medications and hospital-based medical practices, or misinformation. This misinformation can include references to scientifically unfounded and disproven theories, such as the alleged link between vitamin K administration and the development of leukemia [83]. Third, non-administration of vitamin K may occur even in the absence of explicit parental refusal due to structural barriers such as limited drug availability, inadequate healthcare infrastructure, or insufficient training of birth attendants. This scenario is more common in middle- and low-income settings and reflects a system-level challenge rather than an individual parental choice. Additional contributing factors include linguistic and cultural barriers. As demonstrated by the case reported by Elsebey et al. (2024), language-related misunderstandings can lead to inappropriate decisions even when accurate information is provided by healthcare professionals [5]. Finally, this existence regarding IM prophylaxis appears to be more frequent in specific socio-demographic contexts, particularly among mothers with higher educational attainment, belonging to majority ethnic groups, and holding “alternative” views on health and vaccinations [79].

However, this trend is not without consequences. It has already been associated with a resurgence of preventable late VKDB cases, which often present with intracranial hemorrhage, acute anemia, or long-term neurological impairment. A recent study has documented fatal cases or severe sequelae despite intensive treatment [5]. However, the absence of systematic tracking of cases limits the true epidemiological estimation of the phenomenon [83].

Parental hesitancy towards vitamin K prophylaxis represents an emerging public health issue, as documented by Loyal et al. (2017) [78]. This issue is of particular concern in the United States, where the culture of natural childbirth and skepticism towards conventional medicine are widespread. The most recent data from Scott et al. (2025) indicates that this trend is not only persisting but also continuing to increase at a national level. This further underscores the urgent need for coordinated interventions [7].

Communication strategies play a central role in addressing parental hesitancy. Evidence-based communication approaches have been developed to support this process. These include providing anticipatory prenatal counseling, presenting absolute risks and clinical outcomes clearly, concisely rebutting misinformation, and transparently explaining excipients when requested. This approach is preferable to limiting discussions to the immediate postpartum period, which is often high-stress and time-limited. In addition, a respectful “default recommendation” approach, embedded within a shared decision-making framework, may help parents make more informed choices [84].

Moreover, coordinated communication among obstetricians, midwives, and pediatricians, coupled with a clear explanation of the newborn’s biological vulnerability to vitamin K deficiency, may help reduce refusal and support genuinely informed decision-making.

To support clinicians in translating evidence into parent-understandable outcomes, a brief counseling checklist is provided below:

- Rarity versus severity of late VKDB

Late-onset vitamin K deficiency bleeding is rare; however, when it occurs, it is often severe and may present with intracranial hemorrhage, long-term neurological impairment, or death.

- Biological vulnerability of the newborn

Newborns are physiologically predisposed to vitamin K deficiency due to limited placental transfer, low hepatic stores at birth, immature intestinal absorption, and delayed establishment of vitamin K–producing gut microbiota.

- Exclusive breastfeeding and increased risk

Human breast milk contains very low concentrations of vitamin K; therefore, exclusively breastfed infants are at increased risk of VKDB in the absence of effective prophylaxis.

- Intramuscular versus oral prophylaxis

Intramuscular vitamin K provides sustained and reliable protection with a single dose, whereas oral prophylaxis requires strict adherence to multi-dose regimens to be effective.

- Limitations of oral absorption

Conditions such as cholestasis or intestinal malabsorption can significantly impair oral vitamin K absorption, making oral prophylaxis unreliable in these clinical settings.

### Ethical and Legal Considerations Regarding Refusal of Vitamin K Prophylaxis

Refusal of neonatal vitamin K prophylaxis does not represent an individual parental choice solely but has significant public health implications. In recent years, several contributions have addressed the refusal of vitamin K from legal and health policy perspectives. Levy and Nabatian (2025) [77] propose a three-part strategy to address the issue. This includes systematically monitoring refusals and VKDB cases at state and federal levels, implementing evidence-based educational campaigns that clearly explain the benefits and safety of vitamin K, and conducting a regulatory review to harmonize public health policies and define the boundaries of parental autonomy in preventive care more clearly. In a complementary manner, Isennock (2023) [83] has examined the issue in light of child protection principles. In light of the high effectiveness of vitamin K in preventing VKDB and its favorable risk profile, refusal of prophylaxis may, in specific circumstances, be considered a decision that falls within the legal debate on child protection and the definition of medical neglect, given existing precedents in pediatric health law.

Table 3 summarizes the main characteristics of the main studies published between 2014 and 2025 on parental refusal of vitamin K administration at birth, highlighting their geographical distribution, study designs, and clinical and regulatory implications. Overall, the included studies indicated that parental hesitancy of intramuscular vitamin K is a progressively increasing phenomenon, as documented by recent international data principally from the United States (Table 3), and it appears particularly frequent in low-intervention birth settings and in association with hesitancy toward other preventive interventions. Globally, the main barriers to prophylaxis appear to depend on the socioeconomic context: while in high-income countries parental hesitancy predominates, in middle- and low-income settings structural limitations are more prominent. These differences reflect marked heterogeneity among the available studies in terms of geographic setting, study design, and observation period, which contributes to the variability of the reported incidence estimates and should be taken into consideration. Despite this heterogeneity, clinical outcomes are consistently severe, with a high incidence of intracranial hemorrhage and substantial mortality among cases of VKDB. Case reports and ethical–legal analyses further underscore that vitamin K refusal represents an emerging public health issue with significant clinical, communicative, and regulatory implications.

## 10. Future Directions and Research Needs

Although intramuscular vitamin K remains the most reliable prophylactic option, the current body of literature highlights several gaps that warrant targeted future investigation, particularly with respect to oral regimens, genetic determinants, and implementation strategies. Existing evidence on oral administration is heterogeneous, with variation in formulations, dosing schedules, and adherence, making it difficult to determine which strategies can provide consistent long-term protection. In the short term, more rigorous and standardized research should clarify these uncertainties and help identify effective, practical, and parent-acceptable alternatives to IM prophylaxis. There are several potential advantages that could be gained by advancing the study of oral vitamin K. Multi-dose regimens and improved formulations, such as mixed-micellar preparations, could enhance absorption and provide sustained protection, especially when adherence is ensured. Since oral administration avoids injection-related discomfort and rare local reactions, it may increase parental acceptance and reduce refusal rates. Well-designed trials comparing multiple oral schedules could therefore support personalized prophylaxis approaches, better aligned with parental preferences and clinical feasibility. An additional major limitation of the current literature is the lack of studies specifically designed for the neonatal period investigating polymorphisms in the *VKORC1* and *GGCX* genes. In the longer term, further investigation into genetic and developmental determinants of vitamin K metabolism is warranted. Most available evidence is derived from adult populations or from older pediatric cohorts, with neonates being sparsely and often only marginally represented. Moreover, studies directly correlating *VKORC1* and *GGCX* polymorphisms with clinically relevant neonatal outcomes are largely lacking. In particular, it remains unclear whether these genetic variants influence the risk and severity of vitamin K deficiency bleeding (VKDB), including susceptibility to intracranial hemorrhage, or the response to vitamin K prophylaxis, either via the intramuscular or the oral route. At present, these genetic considerations remain primarily research-oriented and are not intended to guide routine clinical practice. Future research should also prioritize long-term follow-up to evaluate late VKDB, identify optimal dosing in high-risk groups (including breastfed or medically fragile infants), and generate pharmacokinetic data that reflect real-world feeding patterns and developmental physiology. Additionally, implementation-focused studies are needed to assess adherence strategies, public health messaging, and system-level supports that can improve the consistency of oral regimen delivery. Overall, strengthening the evidence base for oral vitamin K could expand safe and effective prophylaxis options, support shared decision-making with families, and contribute to more flexible prevention strategies without compromising clinical outcomes.

## 11. Conclusions

Vitamin K prophylaxis remains a cornerstone of neonatal preventive care and has profoundly reduced the incidence of vitamin K deficiency bleeding (VKDB) worldwide. The evidence synthesized in this narrative review confirms that a single intramuscular dose of vitamin K_1_ administered at birth provides the most reliable protection against both classic and late VKDB, with consistently high effectiveness across diverse healthcare settings. Oral prophylactic regimens, while offering a potential alternative when intramuscular administration is declined, show greater variability in efficacy, largely influenced by formulation, dosing schedules, and adherence, particularly in real-world conditions. From a mechanistic perspective, the unique vulnerability of newborns to vitamin K deficiency is rooted in the immaturity of placental transfer, hepatic storage capacity, and intestinal absorption, as well as in the central role of the vitamin K cycle, mediated by γ-glutamyl carboxylase and *VKORC1*, in activating coagulation and other vitamin K-dependent proteins. Integrating these molecular and physiological insights with clinical evidence helps to explain the differential effectiveness of prophylactic strategies and reinforces the rationale for early, reliable supplementation. Beyond efficacy and safety, increasing parental hesitation and refusal of intramuscular vitamin K has emerged as a relevant public health challenge, contributing to preventable cases of VKDB with potentially severe neurological consequences. In the short term, priority should be given to implementation-focused research, improved clinician–parent communication, and surveillance strategies aimed at maintaining high prophylaxis coverage. In the longer term, further investigation into optimized oral formulations, pharmacokinetics, and pharmacogenetic variability may support more personalized preventive approaches. Overall, this review provides an integrated framework linking molecular mechanisms, neonatal physiology, clinical effectiveness, and real-world implementation issues. Such a comprehensive perspective is essential to inform evidence-based practice, guide public health strategies, and ensure sustained protection of newborns from this largely preventable condition.

## Figures and Tables

**Table 1:** Overview of international recommendations for neonatal vitamin K prophylaxis. Guideline-based data summarized from ESPGHAN [26]. Supporting clinical evidence from observational studies and reports [24,25,27].

VKDB Subtype	Typical Timing	Key Risk Factors	Typical Bleeding Sites	Incidence Without Prophylaxis (Cases Per 100,000 Live Births)	Clinical Outcomes	Preventability by Prophylaxis
**Early VKDB**	≤24 h of life	Maternal exposure to vitamin K-antagonist drugs (anticonvulsants, vitamin K antagonists, antituberculosis drugs)	Intracranial, gastrointestinal, abdominal, thoracic	~250–1700	Poor prognosis; intracranial hemorrhage in up to 25% of cases	Limited—neonatal prophylaxis less effective; prevention depends mainly on maternal management
**Classic VKDB**	2–7 days of life	Lack of vitamin K prophylaxis; exclusive breastfeeding	Skin, umbilical stump, injection sites, gastrointestinal tract	~10–35	Usually mild to moderate; CNS involvement rare	Highly preventable with IM or appropriate oral prophylaxis
**Late VKDB**	2–12 weeks (up to 6 months in exclusively breastfed infants)	Lack of prophylaxis; exclusive breastfeeding; cholestasis or malabsorption	Intracranial, gastrointestinal, cutaneous	~10–80	Severe; intracranial hemorrhage common; mortality and long-term neurological sequelae in 40–55% of survivors	Near-completely preventable with universal IM vitamin K

**Table 2:** Existing guidelines on vitamin K prophylaxis in newborns.

Guideline	General Recommendations
**AAP**	Vitamin K should be administered to all newborns who weigh more than 1500 g as a single, intramuscular dose of 1 mg within six hours of birth. Preterm infants with weight ≤ 1500 g should receive a vitamin K dose of 0.3–0.5 mg/kg as a single, intramuscular dose. A single intravenous dose of vitamin K for preterm infants is not recommended for prophylaxis [3].
**WHO**	All newborns should be given 1 mg of vitamin K intramuscularly after birth: after the first hour, during which the infant should be in skin-to-skin contact with the mother and breastfeeding should be initiated. This is a strong recommendation, with moderate-quality evidence. Neonates requiring surgical procedures, those with birth trauma, preterm newborns, and those exposed in utero to maternal medication known to interfere with vitamin K are at especially high risk of bleeding and must be given vitamin K [1 mg IM]. This is a strong recommendation with moderate-quality evidence [38,39].
**NICE**	All parents should be offered vitamin K prophylaxis for their babies to prevent the rare but serious and sometimes fatal disorder of vitamin K deficiency bleeding. Vitamin K should be administered as a single dose of 1 mg intramuscularly, as this is the most clinically and cost-effective method of administration. If parents decline intramuscular vitamin K for their baby, oral vitamin K should be offered as a second-line option. Parents should be advised that oral vitamin K must be given according to the manufacturer’s instructions for clinical efficacy and will require multiple doses. Note: These recommendations were established in 2006 when the first clinical guideline was published and confirmed in 2015. In the latest NICE guidelines (2021), it is not covered, as it is considered an established practice with no changes compared to what was previously stated [40].
**ESPGHAN**	Healthy newborn infants should either receive: - 1 mg of vitamin K1 by IM injection at birth, OR - 3 × 2 mg of vitamin K1 orally at birth, at 4 to 6 days, and at 4 to 6 weeks, OR - 2 mg of vitamin K1 orally at birth and a weekly dose of 1 mg orally for 3 months. The oral route is not appropriate for preterm infants and for newborns who are unwell, have cholestasis or impaired intestinal absorption, or are unable to take oral vitamin K, or those whose mothers have taken medications that interfere with vitamin K metabolism [26].

**Abbreviations:** *AAP*, American Academy of Pediatrics; *ESPGHAN*, The European Society for Paediatric Gastroenterology Hepatology and Nutrition; *NICE*, National Institute for Health and Clinical Excellence; *WHO*, World Health Organization.

**Table 3:** Recent studies on parental refusal of vitamin K in newborns.

Authors (Year)	Country/Geographical Area	Study Design/Method	Main Findings (Refusal Rates and Motivations)	Clinical Consequences
Scott et al. (2025) [7]	United States	Retrospective cohort study; >5 million newborns, 2017–2024	Non-receipt of IM vitamin K increased from 2.92% (2017) to 5.18% (2024) (3.92% overall); higher non-receipt among vaginal deliveries and low-intervention birth settings, suggesting growing parental hesitancy toward preventive care	Lack of prophylaxis is associated with late VKDB rates of 10.5–80 per 100,000 live births, with intracranial hemorrhage is ~50% and mortality up to 20%.
Loyal & Shapiro (2020) [85]	United States	Narrative review of original studies on IM vitamin K refusal and VKDB	Refusal rates ranged from 0 to 3.2% in US hospitals, up to 14.5% in home births and 31% in birthing centers; refusal strongly associated with vaccine hesitancy and preference for “natural” approaches	Without prophylaxis, VKDB incidence estimated at 250–1700/100,000 (early) and 10.5–80/100,000 (late); among reported cases, intracranial hemorrhage common, with mortality ≈20% and neurologic sequelae ≈10%
Sirachainan et al. (2025) [80]	38 countries (Asia 71%, Europe 18%, North America 8%)	International cross-sectional survey of 685 clinicians	Three global clusters were identified: (1) high-income countries → parental refusal of IM administration; (2) middle-income countries → liver diseases; (3) low-income countries → drug unavailability. Mortality: 3.4%; morbidity: 30.9%.	Intracranial hemorrhage (≈ 42%), gastrointestinal (≈20%), cutaneous (≈27%); mortality 3.4%, morbidity ≈ 31%.
Elsebey et al. (2024) [5]	United States (Florida)	Descriptive case report	Refusal due to language barrier and misunderstanding despite medical counseling	Hemorrhagic shock, severe coagulopathy (PT > 300 s, INR > 4), emergency neurosurgical intervention; full recovery after treatment with vitamin K, PCC, and plasma
Isennock (2023) [83]	United States	Legal-ethical review	Analyzes the limits of parental autonomy and the state’s duty to protect the child’s health.	Increase in cases of late VKDB, with intracranial hemorrhage and neurological sequelae (seizures, developmental delay, death in 20% of late VKDB cases).
Öztürk et al. (2023) [81]	Turkey	case report	Case of recurrent VKDB in an untreated newborn; the parents had refused vitamin K.	Hemorrhagic shock due to massive intrathoracic hemorrhage
Levy & Nabatian (2025) [77]	United States	Legal and health policy review	They propose classifying refusal as “medical neglect” and standardizing state regulations, citing legal precedents such as Prince v. Massachusetts.	Vitamin K deficiency bleeding (VKDB), including intracranial hemorrhage, with high mortality in late-onset forms.

## Data Availability

No new data were created or analyzed in this study. Data sharing is not applicable to this article.

## Abbreviations

The following abbreviations are used in this manuscript:

AAP	American Academy of Pediatrics
AI	Adequate Intake
apoA	Apolipoprotein A
apoB-48	Apolipoprotein B-48
apoC	Apolipoprotein C
apoE	Apolipoprotein E
AUC	Area Under the (Concentration–Time) Curve
CI	Confidence Interval
CM	Chylomicron
CR	Chylomicron Remnant
CYP4F2	Cytochrome P450 Family 4 Subfamily F Member 2
ESPGHAN	European Society for Paediatric Gastroenterology, Hepatology and Nutrition
*GGCX*	γ-Glutamyl Carboxylase Gene
Gla	γ-Carboxyglutamic Acid
HDL	High-Density Lipoprotein
HDN	Hemorrhagic Disease of the Newborn
HIF-1α	Hypoxia-Inducible Factor 1 Alpha
ICH	Intracerebral Hemorrhage
IM	Intramuscular
IV	Intravenous
LDL	Low-Density Lipoprotein
LDLR	Low-Density Lipoprotein Receptor
LRP1	LDL Receptor-Related Protein 1
MAPK	Mitogen-Activated Protein Kinase
MK-7	Menaquinone-7
MK-9	Menaquinone-9
NADPH	Nicotinamide Adenine Dinucleotide Phosphate
NASPGHAN	North American Society for Pediatric Gastroenterology, Hepatology and Nutrition
NF-κB	Nuclear Factor κ-light-chain-enhancer of activated B cells
NICE	National Institute for Health and Care Excellence
ODS	Office of Dietary Supplements
PCC	Prothrombin Complex Concentrate
PDGFRB	Platelet-Derived Growth Factor Receptor Beta
PFKFB3	6-Phosphofructo-2-Kinase/Fructose-2,6-Bisphosphatase 3
PIVKA	Proteins Induced by Vitamin K Absence
PIVKA-II	Protein Induced by Vitamin K Absence or Antagonist II
PPP	Pentose Phosphate Pathway
RCT	Randomized Controlled Trial
RR	Relative Risk
VLDL	Very-Low-Density Lipoprotein
VK	Vitamin K
VK1	Vitamin K1
VK2	Vitamin K2
VKCFD	Vitamin K-Dependent Coagulation Factors Deficiency
VKDB	Vitamin K Deficiency Bleeding
*VKORC1*	Vitamin K Epoxide Reductase Complex Subunit 1
WHO	World Health Organization
